# Highlight: Delaying the Call of Death—Genome-Wide Study of Long-Lived Fruit Flies

**DOI:** 10.1093/gbe/evt203

**Published:** 2014-01-03

**Authors:** Danielle Venton



Whatever your luck in life, the consequence of old age is unavoidable. Death visits us all. But despite its universality, the timing of this call varies.

In developed nations, most people can expect to live to 80 or 85. Only about 1–2% of the population celebrates their 100th birthday. Like many traits of interest, longevity is complex—determined by the interactions of our behavior, environment, and genetic background.

Centenarians are not that easy to find, so past studies seeking to link genetic variation to longevity in humans have suffered from small sample sizes. However, a robust, novel approach by researchers at the University of California (UC) Irvine and Cornell University, using fruit flies, may point toward a clearer understanding of genome-wide variations associated with longevity in humans as well. The authors describe their results in *Genome Biology and Evolution* (Burke et al. 2013)*.*

Pools of old chromosomal DNA from the longest surviving 2% of females (analogous to centenarians) was sequenced alongside control DNA from young adult females from the same cohort. The flies were from recombinant lines developed by Anthony Long (a coauthor) and Stuart Macdonald as part of the Drosophila Synthetic Population Resource. By using this experimental mapping population with a defined genetic background, Burke and her team could define regions of the *Drosophila* genome that differed between “normal” and long-living flies more rigorously than what is possible in human genome-wide association studies.

“We found using this approach really narrows down the number of locations genome-wide that are implicated in longevity,” said Molly Burke, a postdoctoral researcher at UC Irvine. “That’s what we felt was a strength of the paper.”

Although this current study did not go so far as to pinpoint specific genes, the authors did identify eight regions that seem important. The areas of the genome that most changed between the two groups tended to be toward the ends or the center of the chromosomes—regions that rarely recombine during meiosis. This suggests, says Burke, that deleterious mutations that affect longevity are able to accumulate in these regions of the genome. This observation harmonizes with the mutation accumulation evolutionary theory of aging, based on the idea that unconditionally deleterious alleles can accumulate if their fitness effects strike only in the postreproductive phase of life.

“This is a result of general significance, I think,” Burke says, “that could be informative in the search for human longevity genes. Perhaps the centromeres and telomeres are somewhere we need to look more carefully.”

Regions of normal recombination that differed significantly between the two populations held genes needed for immune function as well as a gene family (glutathione transferase) involved in oxidative stress response. These findings are perhaps intuitive: a healthy immune system would logically help flies fight pathogenic bacteria and live longer as a result. The glutathione transferase family is part of an enzyme class that scavenges free radicals (reactive oxygen species supposed to damage molecule) created by oxidative stress.

“I think it’s a great experiment,” says David Rand, an evolutionary geneticist from Brown University, who studies mitochondrial–nuclear genome interactions within *Drosophila.* “[The authors found] a logical, empirical approach to studying centenarians. In a way, their major result is a lack of a result.”

The authors agree, writing: “While our gene list is ripe for the validation of candidate longevity genes perhaps the list’s most interesting feature is an absence of genes previously described in the literature.”

A long list has been compiled in the fruit fly genetics literature, presenting about 300 alleles in approximately 150 genes as being important for aging. Burke and her colleagues essentially found no overlap.

“This was surprising,” Burke says. “But the flies that we studied in this experiment are not inbred flies, they’re outbred flies that have been freely mating and recombining for many generations, so they’re genetically diverse and more representative of fruit fly populations in the wild.”

Most of the *Drosophila* aging studies historically have used inbred lab strains to study the effects of mutations in genes. Even though those mutations have an effect on lab strains, they’re unlikely to have large effects in outbred, more natural populations.

“This suggests that to find natural variants important for a phenotype like longevity,” says Burke, “we need to do experiments in outbred populations, whatever the species.”

Anthony Long is currently studying another complex phenotype in fruit flies—sensitivity to chemotherapy. His current approach works, he says, but is incredibly labor intensive: “It's not pretty, we operate by brute force.” The pooling technique used here, he believes, points the way forward when studying traits that draw on multiple genetic factors.

“It suggests some approaches where we could take our lines, combine them, treat the combined population with a chemo drug and then identify the elite individuals that survive that treatment well and then sequence that pool,” he says. “This might be 10 to 50 times more efficient, freeing us up to assay some extremely interesting phenotypes that we can’t right now.”

Burke expects the study to be influential in her research as well.

“Just because we identified these really interesting genes in silico,” she says, “does not mean that we have proven that mutations in these genes affect longevity.”

To find such proof, she is learning to do transgenic experiments in yeast—where one version of a gene can be swapped for another. This may, in time, point the way toward genes affecting human longevity as well.

“Given the limitations of working with human studies,” she says, “researching model systems like this is where I think the frontier lies.”
